# Screening swabs surpass traditional risk factors as predictors of MRSA bacteremia

**DOI:** 10.1186/s12879-018-3182-x

**Published:** 2018-06-11

**Authors:** Guillaume Butler-Laporte, Matthew P. Cheng, Emily G. McDonald, Todd C. Lee

**Affiliations:** 1 Division of Infectious Diseases, Department of Medicine, McGill University Health Centre, 1001 Boulevard Décarie, room E05. 1917, Montreal, Quebec, H4A 3J1 Canada; 2 Division of General Internal Medicine, Department of Medicine, McGill University Health Centre, Montreal, Quebec, Canada; 3 Clinical Practice Assessment Unit, McGill University Health Centre, Montreal, Quebec, Canada

**Keywords:** MRSA, Colonization, Vancomycin, Bacteremia, Statistics

## Abstract

**Background:**

Consideration to add empiric MRSA therapy with vancomycin is a common clinical dilemma. However, vancomycin overuse has important adverse events. MRSA colonization screening is commonly performed for infection control. We hypothesized that in cases of *S. aureus* bacteremia, a score based on patient level factors and MRSA colonization could predict the risk of MRSA infection and inform the need for empiric coverage.

**Methods:**

Using modern machine learning statistical methods (LASSO regression and random forests), we designed a predictive score for MRSA infection based on patient level characteristics, and MRSA colonization as measured by screening done 30 days before infection (30-Day criteria), or at any time before infection (Ever-Positive criteria). Patient factors (age, sex, number of previous admissions, and other medical comorbidities) were obtained through our electronic records.

**Results:**

With random forests, MRSA colonization largely surpassed all other factors in terms of accuracy and discriminatory power. Using LASSO regression, MRSA colonization was the only factor with MRSA infection predictive power with odds ratio of 10.3 (min: 5.99, max: 16.1) and 8.14 (min: 6.01, max: 14.8) for the 30-Day and Ever-Positive criteria, respectively. Further, patient comorbidities were not adequate predictors of MRSA colonization.

**Conclusions:**

In an era of community acquired MRSA, colonization status appears to be the only independent and reliable predictor of MRSA infection in cases of *S. aureus* bacteremia. A clinical approach based on a patient’s known MRSA colonization status and on local susceptibility patterns may be appropriate.

**Electronic supplementary material:**

The online version of this article (10.1186/s12879-018-3182-x) contains supplementary material, which is available to authorized users.

## Background

Methicillin resistant *Staphylococcus aureus* (MRSA) colonization is common in North America [1] and is an important cause of infections including skin and soft tissue, bone and joint, pneumonia and bacteremia. Consideration for empiric MRSA treatment results in patients often being prescribed vancomycin for suspected Gram-positive infections, sometimes days before the final microbiological results are obtained. While this is likely initially appropriate for hemodynamically unstable patients or when the index of suspicion for MRSA is reasonably high, the overuse of vancomycin has a significant potential for harm. It is associated with an increased risk of nephrotoxicity, which may be more common when vancomycin is used in combination with piperacillin-tazobactam [2]. This combination is one of the most commonly used treatments for empiric broad spectrum antimicrobial coverage. Vancomycin-associated nephrotoxicity is associated with an increased length of hospitalization, cost, and an increased risk of mortality [3, 4]. Further, the initial use of vancomycin may delay the timely initiation of beta-lactams, which are superior agents for methicillin sensitive *S. aureus* (MSSA) [5].

Our group and others have previously demonstrated that MRSA screening swab results can be helpful in determining the probability of MRSA bacteremia in patients presenting with staphylococcal bloodstream infection [6]. Other factors associated with MRSA infection such as the presence of chronic wounds, venous catheters, or other in-dwelling medical devices have also been studied in cases of MRSA infection of the bone or joint [7, 8].

We hypothesized that by combining individual patient comorbidities and risk factors along with MRSA screening swab results we could better discriminate between MRSA and MSSA bacteremia using statistical algorithms to develop a clinical prediction rule. This would help to avoid potentially inappropriate empiric use of vancomycin.

## Methods

We conducted a retrospective review of all consecutive adult *S. aureus* bacteremia from April 1, 2010 to April 1, 2015 at the McGill University Health Center (832 beds; 2 hospitals in Montréal, Canada) and obtained their most-recent MRSA screening swab results prior to the blood culture. To prevent patients with multiple positive blood cultures from biasing the results of the study, only the first positive blood culture per patient was included.

Each case of bacteremia was cross-referenced with our electronic medical record to identify associated patient comorbidities in order to calculate the Charlson Comorbidity Index (CCI), a validated scoring system used to assess disease burden and mortality risk [9]. An algorithm was used to calculate the CCI from the ICD-10 codes [10]. We also recorded the number of previous emergency room and inpatient visits within the prior 12 months to serve as a proxy for acute healthcare exposure. All patients on medical, surgical and critical care units are screened for MRSA on admission, and periodically thereafter. A minority of units, such as obstetrics, use only targeted screening. The screen is performed in the nares, though other sites are accepted on a case by case basis. The microbiology protocol has been previously described in detail [6].

MRSA colonization status was interpreted in two ways. To begin, only samples obtained in the 30 days preceding the bacteremia were used to establish recent colonization. Following this, all available screening samples in the patient’s history of hospitalizations were used. These were respectively reported as the “30-Day criteria” and the “Ever-Positive criteria” [6].

We used the following two methods for variable selection to predict MRSA bacteremia: [1] random forests methods, and [2] least absolute shrinkage selection and selection operator (Lasso) with 10-fold cross validation and the “one standard error rule” (1SE) [11]. Both methods have their own benefits and pitfalls. Random forests yield good error rates and classifies variables by their accuracy and Gini index (a measure of a variable’s ability to discriminate between the potential outcomes), but are harder to interpret. The Lasso provides a reasonable middle ground between interpretability and stability of the effect size estimate [11]. Other variable selection methods exist; however, they tend to be more susceptible to issues involving multiple hypothesis testing and overestimation of effect size (e.g. stepwise regression) or are difficult to interpret clinically (e.g. ridge regression, neural networks). A secondary analysis using Lasso logistic regression was performed to study the variables that most closely predicted MRSA colonization.

Variables were checked for collinearity using variance inflation factors. Analyses were performed in R (v3.2.0) with the glmnet (v2.0–5) and the randomForest (v4.6–12) packages. The McGill University Health Centre Research Ethics Board approved this study. Consent was not obtained from patients as this was a retrospective study with no intervention. Authorization to release datasets analysed during the current study was not specifically obtained from our ethics board, but can be made available from the corresponding author on reasonable request and in compliance with our REB requirements.

## Results

**Fig. 1:**
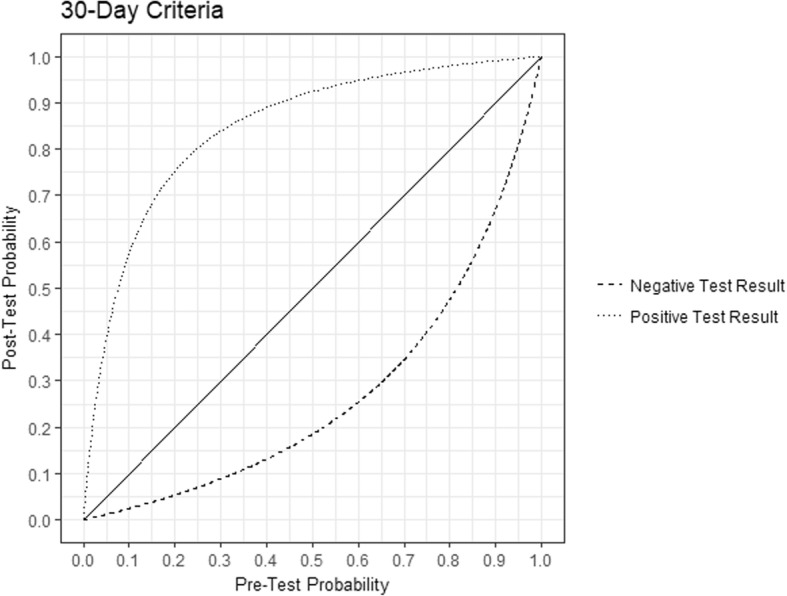
Post-test probability of the 30-Day criteria, as a function of pre-test probability

**Fig. 2:**
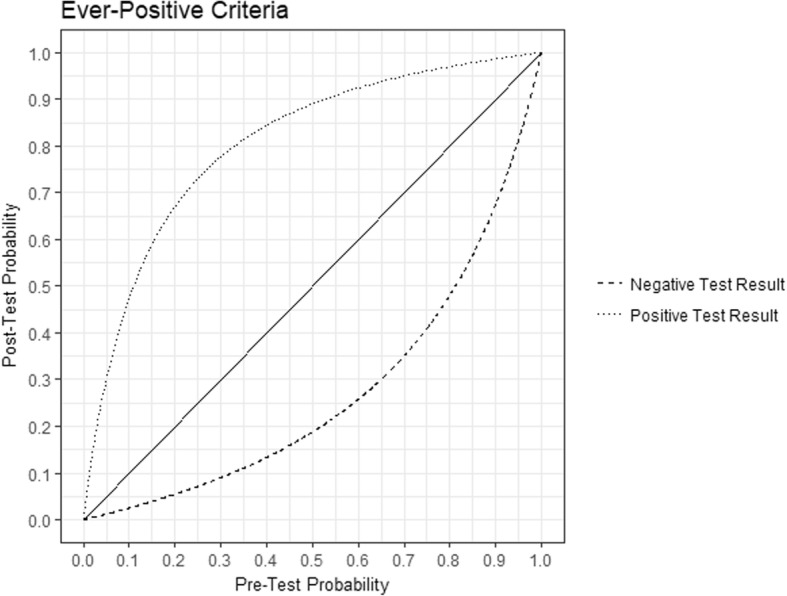
Post-test probability of the Ever-Positive criteria, as a function of pre-test probability

There were 376 patients with *S. aureus* bacteremia included, of which 100 (26.6%) had MRSA. Patient comorbidities are shown in Table 1. Using Random Forest analysis, the five best variables for predicting MRSA bacteremia are presented in Tables 2 and 3. With both the 30-day and the Ever-Positive criteria, MRSA swabs had a predictive power that was vastly greater than all other variables. For example, a positive MRSA swab had a much greater predictive power than the presence of other seemingly classic risk factors such as age, a history of malignancy, the number of prior hospitalizations in the preceding 12 months, and the Charlson Comorbidity Index. Using the Lasso method and1SE rule (see Additional file 1), the MRSA screening swab was the only variable with any clinically relevant predictive power. Through this method, the logistic regression odds ratios for the 30-Day criteria were on average 10.6 (minimum: 5.08, maximum: 19.2), and for the Ever-Positive criteria on average 8.26 (minimum: 5.23, maximum: 15.8). Using Lasso logistic regression, no patient level comorbidity was predictive of MRSA colonization. This suggests the screening swab is likely the *only* meaningful variable in the model with respect to clinical prediction. From the 2 × 2 tables, we obtained a graphical representation of MRSA swab positive and negative predictive values for MRSA bacteremia (Figs 1 and 2) as functions of prevalence.

**Table 1:** Number of patients with each comorbidities (percentage), and median number of admissions to the emergency or a medical ward (interquartile range)

Variables	MSSA (*n* = 276)	MRSA (*n* = 100)	*p*-value (Fisher’s or Mann-Whitney U)
Positive MRSA Screen (30 days)	10/154 (6.49%)	52/66 (78.8%)	< 0.001
Positive MRSA Screen (All-Time)	21/215 (9.77%)	72/91 (79.1%)	< 0.001
Median Age (IQR)	65.5 (51, 76.25)	66 (54.75, 77)	0.590
Median CCI (IQR)	3 (1, 5)	3 (2, 4)	0.155
Myocardial Infarction	39 (14.1)	19 (19)	0.260
Congestive Heart Failure	54 (19.6)	21 (21)	0.771
Peripheral Vascular Disease	31 (11.2)	19 (19)	0.059
Cerebrovascular Disease	18 (6.52)	7 (7)	0.819
Dementia	16 (5.80)	5 (5)	1
Chronic Pulmonary Disease	53 (19.2)	20 (20)	0.883
Connective Tissue Disease	5 (1.81)	5 (5)	0.139
Peptic Ulcer Disease	13 (4.71)	4 (4)	1
Mild Liver Disease	32 (11.6)	9 (9)	0.576
Moderate or Severe Liver Disease	17 (6.20)	4 (4)	0.612
Diabetes without End-Organ Damage	80 (29.0)	36 (36)	0.208
Diabetes with End-Organ Damage	17 (6.16)	10 (10)	0.257
Hemiplegia	12 (4.35)	9 (9)	0.123
Moderate or Severe Renal Disease	5 (1.81)	15 (15)	0.238
Tumor without Metastasis	34 (12.3)	20 (20)	0.068
Tumor with Metastasis	27 (9.78)	8 (8)	0.691
Leukemia	16 (5.80)	6 (6)	1
Lymphoma	21 (7.61)	9 (9)	0.669
HIV/AIDS	3 (1.09)	0 (0)	0.568
Emergency Visits in Last 6 Months	1 (1, 2)	1 (1, 3)	0.189
Emergency Visits in Last 12 Months	1 (1, 2)	2 (1, 3)	0.258
Inpatient Admissions in Last 6 Months	0 (0, 1)	1 (0, 1)	0.003
Inpatient Admissions in Last 12 Months	0 (0, 1)	1 (0, 2)	0.001

**Table 2:** Best 5 variables when using random forests with 30-Day criteria, from best to worse

Variables	Mean Increase in Accuracy (IQR)	Variables	Mean Decrease in Gini (IQR)
30-Day criteria	73.4 (72.2–75.0)	30-Day Criteria	35.9 (35.7–36.2)
Tumor without Metastasis	10.9 (10.4–11.5)	Age	9.14 (9.05–9.24)
CCI	10.2(9.63–10.9)	CCI	6.69 (6.61–6.77)
Congestive Heart Failure	6.57 (5.94–7.32)	Admissions (12 Months)	5.10 (5.03–5.16)
Dementia	6.20 (5.64–6.78)	Myocardial Infarction	2.06 (2.03–2.10)

**Table 3:** Best 5 variables when using random forests with Ever-Positive criteria, from best to worse

Variables	Mean Increase in Accuracy (IQR)	Variables	Mean Decrease in Gini (IQR)
Ever-Positive Criteria	83.2 (82.2–84.5)	Ever-Positive Criteria	45.5 (45.2–45.9)
Tumor without Metastasis	15.6 (15.2–16.1)	Median Age	13.3 (13.1–13.4)
Tumor with Metastasis	9.87 (9.42–10.3)	Admissions (12 Months)	8.17 (8.10–8.30)
CCI	9.76 (8.98–10.1)	CCI	7.97 (7.89–8.07)
Diabetes without End-Organ Damage	9.39 (8.75–9.94)	Diabetes without End-Organ Damage	2.83 (2.79–2.88)

## Discussion

Vancomycin overuse is common in *S. aureus* bacteremia and may be harmful. Our group and others have previously shown that screening swabs are useful predictors of MRSA bacteremia [6, 12], and could help avoid unnecessary vancomycin prescriptions. However, it is a common belief that MRSA colonization status can be explained by healthcare exposure or the presence of multiple medical comorbidities, and that the swab is a proxy for these comorbidities. Previous studies have discussed several potential predictors of MRSA infection [7, 8], but the added benefit of MRSA colonization for clinical decision making was unclear. We examined multiple patient level factors to try and determine which, if any, could predict methicillin resistance, in an effort to develop a clinically meaningful risk score to better optimize the use of anti-MRSA therapy. Our results suggest screening swabs are an independent marker of MRSA infection, regardless of patient comorbidities. The findings of our study are a departure from what has been previously quoted in the literature, in that the only patient level risk factor with a useful clinical predictive ability was a patient’s known MRSA status. In fact, the burden of comorbid disease as calculated by the CCI was poorly related to methicillin resistant bacteremia, and both emergency department visits and admissions to acute care failed to contribute significantly.

This outcome may be related to two factors. First, our choice of variable selection technique prioritizes *clinical* significance over *statistical* significance. This would explain why variables which were previously reported as statistically related to MRSA did not significantly contribute to predicting infections in our study. Second, over the past 20 years MRSA has become a community acquired organism, and hospital exposure or medical comorbidities may not predict infection risk as well as they once might have. Even patients with minimal healthcare exposure may now be carriers of MRSA.

Three main problems limit our study. First, there were a small number of patients with each individual comorbidity in Table 1. To alleviate the problem of smaller sample size, we used an established comorbidity score (CCI) and input direct healthcare exposure as separate variables in our model. This continued to support the use of the MRSA screening swab as the sole predictor of MRSA bacteremia. Second, this study is retrospective and therefore hypothesis generating as to whether the use of known MRSA status could optimize vancomycin use with neutral or improved patient outcomes. Third, our institution only regularly screens for MRSA of the nares. While the nares are the most common site for MRSA colonization and correlates well with colonization of other body sites [13], the addition of other body sites would have improved our test sensitivity, though the incurring loss of specificity is hard to establish. However, it is likely the overall effect would be an improvement of the negative predictive value with a reduction in the positive predictive value. Given the simplicity of the clinical question, and the fact that the data required for the analysis can be collected retrospectively without introducing significant bias, we still believe that our results could be helpful for clinicians who want to estimate the risk of MRSA bloodstream infection.

First, depending on the local prevalence of methicillin resistance in *S. aureus* bacteremia and in the absence of a nosocomial MRSA outbreak, MRSA colonization status could be factored into the clinical decision to initiate intravenous vancomycin in stable patients and antistaphylococcal beta-lactams in all patients so as to limit both under and overtreatment [6]. Second, we should reconsider whether MRSA colonization status is directly linked to the usual medical comorbidities. Based on our results, variables that were previously thought to be associated with MRSA such as healthcare exposure may not be dependable factors to infer the presence of drug resistant bacteria. The notion that healthcare exposure is a consistently reliable marker for the presence of resistant organisms is being challenged, as was illustrated in recent IDSA guidelines with the removal of the classification of “Healthcare-Associated” pneumonia [14]. In an era of community acquired resistant organisms such as MRSA, our results suggest that favoring an approach based on known colonization status and local susceptibility patterns may be more appropriate.

## Conclusions

We have demonstrated that known MRSA colonization status, along with knowledge of local prevalence patterns, is a more powerful prediction tool than any other patient level comorbidity in guiding early and appropriate treatment in *Staphylococcus aureus* bacteremia. We hope that prospective studies will demonstrate that in appropriately selected hemodynamically stable patients this approach reduces the overuse of potentially toxic therapies, increases early targeted beta-lactam therapy, and improves patient outcomes.

## Additional file

Additional file 1: Lasso Analysis An example of the data analysis performed using the Lasso method. The following are representative curve obtained from repeating the analysis 100 times. **Figure S1A.** Patient level comorbidities and 30-Day criteria vs MRSA bacteremia. **Figure S1B.** Patient level comorbidities and Ever-Positive criteria vs MRSA bacteremia (DOCX 2607 kb).

## Abbreviations

1SE: 1 standard error rule
CCI: Charlson comorbidity index
MRSA: Methicillin resistant *Staphylococcus aureus*
MSSA: Methicillin sensitive *Staphylococcus aureus*
