# Supplementary material for: Screening swabs surpass traditional risk factors as predictors of MRSA bacteremia
Source: BMC Infect Dis. 2018 Jun 11;18:270. doi: 10.1186/s12879-018-3182-x (PMC5996459; doi:10.1186/s12879-018-3182-x)
Supplement: Supplementary file 1 — Lasso Analysis An example of the data analysis performed using the Lasso method. The following are representative curve obtained from repeating the analysis 100 times. Figure S1A. Patient level comorbidities and 30-Day criteria vs MRSA bacteremia. Figure S1B. Patient level comorbidities and Ever-Positive criteria vs MRSA bacteremia (DOCX 2607 kb). [file 12879_2018_3182_MOESM1_ESM.docx]

**Supplement 1**

Mean squared error (MSE) as a function of the Lasso coefficient Lambda (here on log scales). The larger the Lambda, the more shrinkage, and the less variables are selected. Number of variables selected are on top the of plots. The dashed vertical line on the left corresponds to the minimum MSE obtained. The dashed line on the right corresponds to the 1SE rule as suggested by Tibshirani et al.

The following are representative curve obtained from repeating the analysis 100 times.


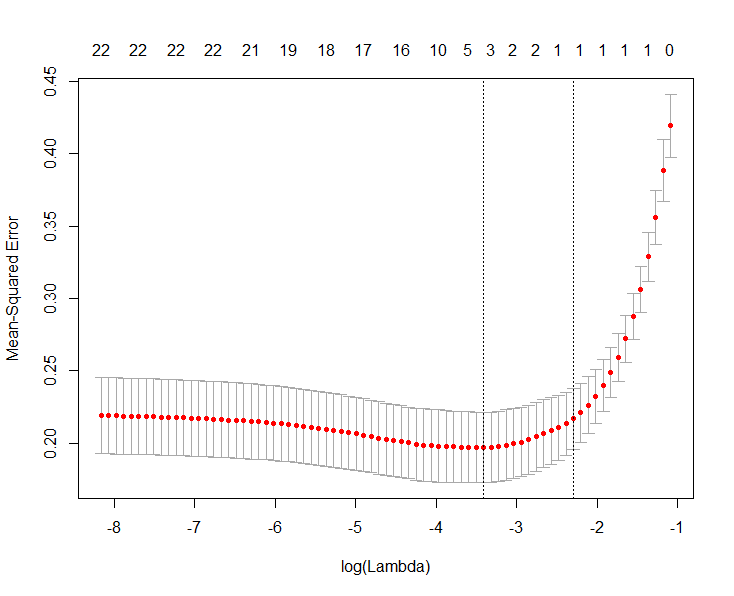


S1a : Patient level comorbidities and 30-Day criteria vs MRSA bacteremia


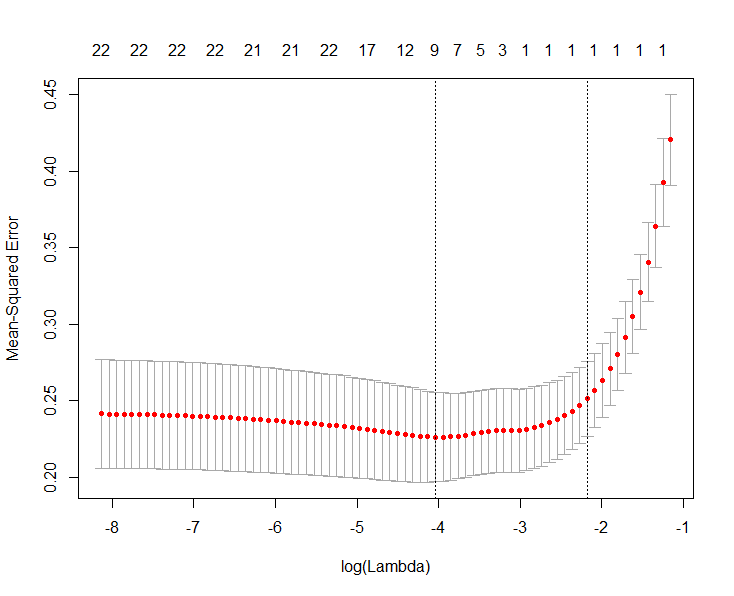


S1b : Patient level comorbidities and Ever-Positive criteria vs MRSA bacteremia
